# The CADILLAC risk score accurately identifies patients at low risk for in-hospital mortality and adverse cardiovascular events following ST elevation myocardial infarction

**DOI:** 10.1186/s12872-021-02348-0

**Published:** 2021-11-12

**Authors:** Ryan S. Wilson, Peter Malamas, Brent Dembo, Sumeet K. Lall, Ninad Zaman, Brandon R. Peterson

**Affiliations:** Department of Medicine, Penn State College of Medicine, Penn State Milton S. Hershey Medical Center, 500 University Drive, Box H047, Hershey, PA 17033 USA

**Keywords:** ST segment elevation myocardial infarction, Coronary artery disease, Risk stratification

## Abstract

**Background:**

The CADILLAC risk score was developed to identify patients at low risk for adverse cardiovascular events following ST elevation myocardial infarction (STEMI) treated with primary percutaneous coronary intervention (PPCI).

**Methods:**

We performed a single center retrospective review of STEMI hospitalizations treated with PPCI from 2014 to 2018. Patients were stratified using the CADILLAC risk score into low risk, intermediate risk and high risk groups. Patients presenting with cardiac arrest or cardiogenic shock were excluded from the study. The primary outcome was adverse clinical events during initial hospitalization. Secondary outcomes were adverse clinical events at 30 days and 1 year following index hospitalization.

**Results:**

The study included 341 patients. Compared to patients with a low CADILLAC score, adverse clinical events were similar in the intermediate risk group during hospitalization (OR 1.23, CI 0.37–4.05, *p* 0.733) and at 30 days (OR 2.27, CI 0.93–5.56, *p* 0.0733) while adverse clinical events were significantly elevated in the high risk group during hospitalization (OR 4.75, CI 1.91–11.84, *p* 0.0008) and at 30 days (OR 8.73, CI 4.02–18.96, *p* < 0.0001). At 1 year follow-up, compared to the low risk CADILLAC group (9.4% adverse clinical event rate), cumulative adverse clinical events were significantly higher in the intermediate risk group (22.9% event rate, OR 2.86, CI 1.39–5.89, *p* 0.0044) and in the elevated risk group (58.6% event rate, OR 13.67, CI 6.81–27.43, *p* < 0.0001). The mortality rate was 0% for patients defined at low risk by CADILLAC score during hospitalization, as well up to 1 year follow up. On receiver operating curve analysis, discrimination of in-hospital adverse clinical events was fair using CADILLAC (C = 0.66, odds ratio 1.18; 95% CI 1.04–1.33; *p* = 0.0064) with somewhat better discrimination at 30-day follow-up (C = 0.719) and 1-year follow-up (C = 0.715).

**Conclusion:**

Patients defined as low risk by the CADILLAC score following a STEMI were associated with lower mortality and adverse clinical event rates during hospitalization and up to 1 year following STEMI when compared to those with an intermediate or high CADILLAC score.

## Introduction

Primary percutaneous coronary intervention (PPCI) has become the cornerstone for management of patients presenting with ST elevation myocardial infarctions (STEMI). When compared to fibrinolysis, PPCI has shown significant reductions in morbidity, mortality, and reduced risk of mechanical and arrhythmic complications [1–12]. Given the improvement in outcomes with PPCI, interest has been placed on the ability to further risk stratify patients presenting with STEMI and identify those at highest risk for complications and mortality. The CADILLAC risk score is one of many scoring systems which was developed to further risk stratify this patient population. This risk score evaluates several different prognostic variables including: Left Ventricular Ejection Fraction (LVEF), Creatinine Clearance, Killip Class, Final Thrombolysis in Myocardial Infarction (TIMI) flow, Age, Anemia, presence of three vessel disease. Utilizing the CADILLAC risk score patients were stratified by low (CADILLAC score 0–2), intermediate (CADILLAC score 3–5), and high (CADILLAC score ≥ 6) risk groups [13]. A previous single center study evaluating 228 total patients presenting with a STEMI, suggested a low event rate and zero percent mortality rate in patient with a low CADILLAC risk score when excluding patients presenting with cardiac arrest or need for mechanical support [14]. We sought to further validate the CADILLAC risk score in patients presenting with a STEMI without cardiac arrest, cardiogenic shock, or need for mechanical support on admission, and determine its ability to risk stratify STEMI patients during their index hospitalization, as well up to 1 year following initial presentation.

## Methods

Institutional Review Board (IRB) approval was obtained for the study. We conducted a single center retrospective review at an academic medical center of all adult patients who presented with a STEMI over a 4-year time period (January 1, 2014 to December 31, 2018). Patients presenting with cardiogenic shock, cardiac arrest, or need for mechanical circulatory support on admission were excluded from the study. The medical records of all patients were comprehensively and systematically reviewed, including physician notes, imaging data, laboratory data, and medication records. All clinical events were recorded and reviewed. Study data was collected and managed using REDCap electronic data capture tools hosted at Penn State Medical Center. REDCap (Research Electronic Data Capture) is a secure, web-based application designed to support data capture for research studies, providing: (1) an intuitive interface for validated data entry; (2) audit trails for tracking data manipulation and export procedures; (3) automated export procedures for seamless data downloads to common statistical packages; and (4) procedures for importing data from external sources [15].

Baseline demographic data was obtained from the chart at time of initial hospital presentation. Cardiac catheterization data was obtained from reports written by interventional cardiology team. Culprit lesion was determined by interventional cardiology team at time of procedure and documented in report. Non-culprit coronary vessels were considered to have significant occlusive disease if documented as greater than 70% stenosis or FFR/iFR positive on invasive hemodynamic assessment. Laboratory values were based on blood work taken on admission. The overall cardiac function was determined by ejection fraction noted on transthoracic echocardiogram which was performed during the hospitalization (1–2 days following intervention). The hospital charts, discharge summaries, and outpatient cardiology notes were reviewed for each individual patient to determine adverse clinical events.

The CADILLAC risk score was calculated for each individual patient. The scoring system was calculated based on admission characteristics, coronary angiography data, and echocardiogram data during index hospitalization. The CADILLAC score assigned a point value to each of seven prognostic variables which included: Baseline LVEF < 40% (4 points), renal insufficiency with Glomerular Filtration Rate (GFR) < 60 (3 points), Killip class 2,3 (3 points), final TIMI flow 0–2 (2 points), age > 65 years (2 points), anemia; defined as hematocrit < 36% in females and < 39% in males (2 points), 3 vessel disease (2 points). Additive scores for each individual were calculated between 0 and 18. Based on definitions used in previous trials [13, 14]; a score of 0–2 was considered “low risk”, a score of 3–5 was considered “intermediate risk”, and a score of 6 or greater considered “high risk”. Patients were grouped accordingly into a low risk, intermediate risk, and high risk cohorts (Table 1).

**Table 1:** CADILLAC risk score

CADILLAC Risk Score	
Score > 5 High	
Score 3–5 Intermediate	

Score 0–2 Low	
Risk Factors	Score
Baseline LVEF < 40%	4
Cr clearance < 60 mL/min	3
Killip class II/III	3
Final TIMI flow 0–2	2
Age > 65 years	2
Anemia (Hgb < 13.0 mg/dL (males) or < 12.0 mg/dL (females)	2
Three vessel disease	2

The primary outcome evaluated was mortality and major adverse cardiovascular events (MACE) during the index hospitalization. MACE was defined as: sustained ventricular tachycardia or fibrillation; recurrent chest pain or ischemia on non-invasive testing which led to repeat coronary angiography; congestive heart failure requiring intravenous diuretic therapy; and stroke/transient ischemic attack documented by neurology team. The secondary outcome evaluated cumulative mortality and MACE at 30-day follow up and 1-year follow up. Mortality and MACE were compared between patients with low risk CADILLAC score (0–2) versus those with intermediate risk scores (3–5) and high risk scores (≥ 6).

Cumulative event rates were tabulated for 30-day and 1-year follow up for each group. Event rates for each group were based off the number of patients from the initial hospitalization and did not change based on percentage of patients who followed up. Odds ratios were calculated to compare the intermediate risk and high risk group event rates to the low risk cohort. Odds ratios were presented with 95% confidence intervals (CI) and p values. Statistically significant p values were considered if < 0.05. Receiver operating characteristic analysis was used to assess use of the CADILLAC risk score to discriminate mortality and MACE during the index hospitalization as well as at 30 day follow up and 1 year follow up.

## Results

Over a 4-year period (from January 1, 2014 to December 31, 2018), a total of 441 patients presented with an STEMI. Of studied patients, 100 individuals at time of presentation had cardiac arrest, cardiogenic shock, or need for mechanical circulatory support on admission and were excluded from the study (22.7%). Of the remaining 341 patients included in the study, 213 had a low risk CADILLAC score (0–2), while 70 had an intermediate risk score (3–5), and 58 had a high risk score of 6 or higher. Table 2, shows a comparison of the medical history and clinical risk factors along with cardiac catheterization and echocardiographic findings for the low, intermediate and high risk groups. The median CADILLAC score in the low risk group was 0.78, compared to 4.0 in the intermediate group, and 8.0 in the high risk group.

**Table 2:** Baseline patient characteristics by CADILLAC risk group

Characteristic	CADILLAC Score^a^ < = 2 (n = 213)	CADILLAC Score^b^ 3–5 (n = 70)	CADILLAC Score^c^ > = 6 (n = 58)
	n (%)	n (%)	n (%)
**Medical history**			
Age (years)	57.6	65.1	69.0
Male	161 (75.6)	53 (76.8)	41 (70.7)
HTN	112 (52.8)	43 (62.3)	48 (82.8)
HLD	78 (36.6)	32 (46.4)	40 (69.0)
DM	38 (17.8)	18 (25.7)	25 (43.1)
Insulin use	7 (18.4)	6 (35.3)	8 (32.0)
Current Smoking	103 (48.4)	25 (36.2)	13 (22.4)
Anemia	20 (9.4)	21 (30.0)	35 (60.3)
CKD (GFR < 60)	0 (0.0)	16 (23.2)	19 (32.8)
Dialysis	0 (0.0)	0 (0.0)	2 (11.1)
Previous CAD	34 (16.0)	18 (25.7)	28 (48.3)
**Killip Class**^d^			
I	208 (98.6)	56 (80.0)	40 (69.0)
II	0 (0)	11 (15.7)	12 (20.7)
III	0 (0)	2 (2.9	4 (6.9)
IV	3 (1.4)	1 (1.4)	2 (3.4)
**TTE LVEF**	55	50	38
**Catherization**			
Multivessel Dx	76 (35.7)	25 (35.7)	36 (62.1)
Final TIMI Flow	2.93	2.91	2.62
*Culprit Lesion*			
Left main	3 (1.4)	0 (0.0)	0 (0)
LAD (or branch off)	73 (34.3)	30 (42.9)	29 (50.0)
LCx (or branch off)	31 (14.6)	8 (11.4)	7 (12.1)
RCA	105 (49.3)	30 (42.9)	22 (37.9)
Ramus	1 (0.5)		0 (0)
Other or none	2 (0.9)	2 (2.9)	0 (0)

^a^Low Risk group

^b^Intermediate Risk group

^c^High Risk group

^d^Killip Class at presentation

In hospital MACE events were seen less frequently in patients with a low CADILLAC score (4.7%), compared to an intermediate score (5.7%) and a high risk score (19%) (Table 3). Nine individuals had 10 adverse events in the low risk group, compared with 12 individuals having 15 adverse events between the intermediate and high risk groups. There were no in-hospital deaths in the low risk CADILLAC group, compared to 1 death in the high risk group. Odds ratios were calculated comparing events in the intermediate or high CADILLAC cohorts to patients with a low risk CADILLAC score (Table 3). Odds ratio demonstrates that patients with a high risk CADILLAC score (OR 4.75, CI 1.91–11.84, *p* 0.0008) had a statistically significant higher chance of having an adverse event during their index hospitalization. Those with an intermediate CADILLAC score did not have a statistically significant difference (OR 1.23, CI 0.37–4.05, *p* 0.7333).

**Table 3:** Cumulative adverse cardiovascular events

	In Hospital Events			30 Day Follow-Up Events			1 Year Follow-Up Events		
	CADILLAC Score < = 2	CADILLAC Score 3–5	CADILLAC Score > = 6	CADILLAC Score < = 2	CADILLAC Score 3–5	CADILLAC Score > = 6	CADILLAC Score < = 2	CADILLAC SCORE 3–5	CADILLAC Score > = 6
n of patients	213	70	58	172	53	48	154	46	40

Complications	n (%)	n (%)	n (%)	n (%)	n (%)	n (%)	n (%)	n (%)	n (%)
VF/VT	3 (1.4)	1 (1.4)	4 (6.9)	3 (1.4)	1 (1.4)	4 (6.9)	3 (1.4)	1 (1.4)	5 (8.6)
MI or UA^a^	2 (0.9)	1 (1.4)	1 (1.7)	4 (1.9)	2 (2.9)	3 (5.2)	10 (4.7)	5 (7.1)	6 (10.3)
CHF	3 (1.4)	2 (2.9)	4 (6.9)	4 (1.9)	5 (7.1)	10 (17.2)	5 (2.4)	9 (12.9)	19 (32.8)
Stroke	2 (0.9)	0 (0.0)	1 (1.7)	2 (0.9)	0 (0.0)	1 (1.7)	2 (0.9)	0 (0.0)	1 (1.7)
Death	0 (0.0)	0 (0.0)	1 (1.7)	0 (0.0)	1 (1.4)	3 (5.2)	0 (0.0)	1 (1.4)	3 (5.2)
Total Events	10 (4.7)	4 (5.7)	11 (19.0)	13 (6.1)	9 (12.9)	21 (36.2)	20 (9.4)	16 (22.9)	34 (58.6)

^a^Recurrent MI or Unstable Angina

**Fig. 1:**
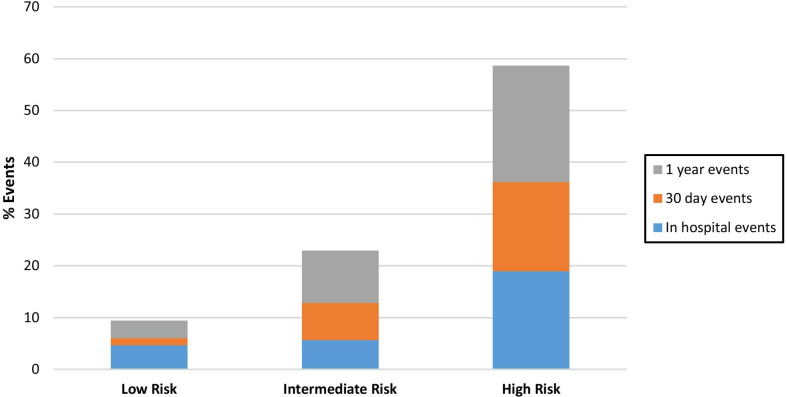
Percentage of adverse clinical events and death in STEMI following PCI, stratified by CADILLAC Risk Score

The secondary analysis involved cumulative MACE and mortality rates at 30-day and 1-year follow-up. At 30-day follow-up, patient in the low risk CADILLAC group had less cumulative adverse events (6.1%) compared to those in the intermediate (12.9%) and high risk group (36.2%) (Table 3). Compared to the low risk group, the risk of adverse clinical events at 30 days was not significantly elevated in the intermediate risk group (OR 2.27, CI 0.93–5.56, *p* 0.0733), but was significant in the high risk group (OR 8.73, CI 4.02–18.96, *p* < 0.0001). Cumulatively, 12 patients had 13 adverse events in the low risk group compared to 21 patients having 30 adverse events in the intermediate-high risk group. No mortality events were seen in the low risk CADILLAC group at 30 days. The cumulative rise of adverse clinical events at 30-days compared to hospitalization in low risk group was 1.4%, compared to 7.2% in the intermediate group and 17.2% in the high risk group (Fig. 1). The rise in mortality rates in the intermediate risk and high risk groups were 1.4% and 3.5%, respectively.

At 1-year follow-up, patients in the low risk CADILLAC group had less cumulative adverse events (9.4%) compared to the intermediate risk group (22.9%) and the high risk group (58.6%) Compared to the low risk group, the risk of adverse clinical events at 1 year was significantly elevated in the intermediate risk group (OR 2.86, CI 1.39–5.89, *p* 0.0044), and the high risk group (OR 13.67, CI 6.81–27.43, *p* < 0.0001). The cumulative rise in adverse events at 1-year compared to hospitalization in the low risk group was 4.7%, compared to 17.2% and 39.6% in the intermediate and high risk groups, respectively (Fig. 1). Overall, 19 total patients had 20 adverse events in the low risk CADILLAC group compared with 34 patients having 50 total events combined in the intermediate and high risk group.

Patients with a low risk CADILLAC group had 0 deaths across out to 1 year follow up in the study cohort (Table 3), while there was 1 death at 30 day follow up in the intermediate risk group. In the high risk group there was one death during time of hospitalization and 2 additional deaths at 30 day follow up. Follow-up retention was similar between all three groups.

**Fig. 2:**
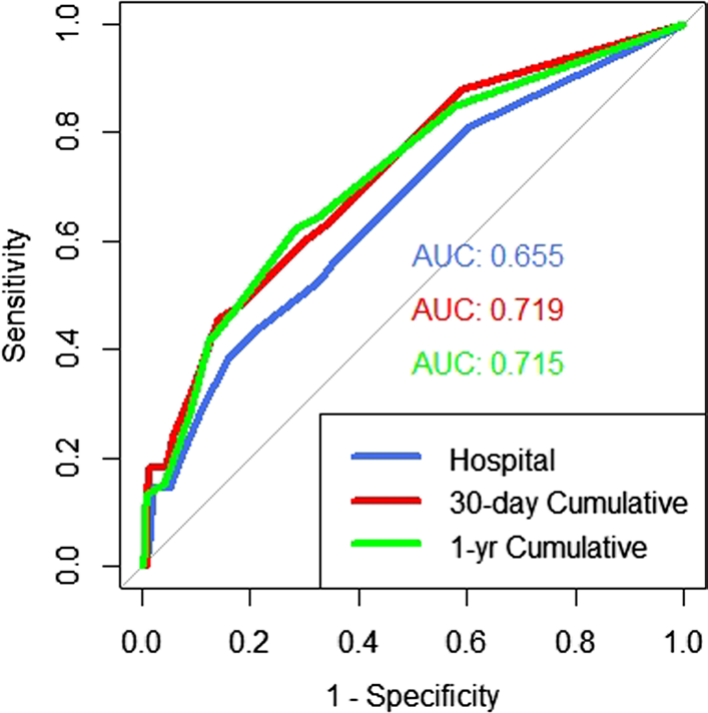
CADILLAC risk score predicting adverse events

The ability of the CADILLAC risk score to predict in-hospital adverse events was calculated by ROC curve (C = 0.66, odds ratio 1.18; 95% CI 1.04—1.33; *p* = 0.0064). The CADILLAC score prediction accuracy improved at 30-day (C = 0.719) and remained consistent at 1-year (C = 0.715), Fig. 2.

## Discussion

The CADILLAC risk score was developed to identify patients at low risk for adverse cardiovascular events following a STEMI treated with PPCI. A previous study evaluating the CADILLAC score found that individuals with a score of ≤ 2 had a lower clinical event rate within the first 24 h post STEMI, as well, a lower event rate on day 3 or later of the index hospitalization, when compared to those with a CADILLAC score of 3 or higher [13, 14].

The results of our study further expanded upon the utility of the CADILLAC risk score and further validated the scoring system as a viable option for identifying patients at low risk for adverse cardiovascular events following a STEMI during their hospitalization. Based on our study, patients with a low CADILLAC score (2 or less) had a significantly lower adverse event rate (MACE + mortality) than those with a high CADILLAC score. Patients with an intermediate risk score although not a statistically significant difference did trend towards a higher event rate (OR 1.23). Also, importantly there were no deaths in the low risk CADILLAC group. The in-hospital event rate in our study compared similarly to the data seen in previous studies evaluating the CADILLAC risk score which had an event rate within the first 24 h of 3.3% vs. 13.3% when comparing the two separate groups. As well, the previous study demonstrated a zero percent mortality rate in the low risk group as well [13, 14].

This study included 213 patients identified as low risk (CADILLAC ≤ 2), a significantly larger sample size then the previous study sample size which included 123 low risk patients [13, 14]. Even with the larger sample size, a similar in-hospital event rate was seen in the low risk group. Additionally, as seen in the previous study, there were no deaths noted in the low risk group during the index hospitalization. Similar patient characteristics were noted between the two studies. Patients with a low CADILLAC risk score had fewer cardiovascular risk factors, no incidence of chronic kidney disease, predominantly presented with Killip class I symptoms, had a normal ejection fraction post revascularization, less likely to have LAD vessel as the culprit lesion, and less likely to have multi-vessel coronary artery disease.

This study sought to further expand on the utility of the CADILLAC risk score and determine if the scoring system was a predictor of events not just during the index hospitalization, as previously studied, but able to predict events at both 30 days and 1 year following the index event. We found that patients with a low CADILLAC risk score had significantly lower cumulative clinical events and mortality compared to the high risk cohort at both 30 days and 1 year. Most importantly zero deaths were noted out to 1 year in patients with a low CADILLAC risk score. Patients with an intermediate CADILLAC score (3–5) at 30 days trended towards an increased event rate when compared to the low risk cohort, however did not meet statistical significance (*p* = 0.0733). At 1 year there was a statistically significant event rate comparing the intermediate risk cohort to the low risk cohort.

Based on these findings, we were able to further support the ability of the CADILLAC risk score to distinguish those patients whom are at lowest risk of complications following a STEMI both during hospitalization and up to 1 year follow up. It is important to note that this study as well as previous studies excluded all patients whom presented with cardiogenic shock, cardiac arrest, or need for ionotropic support. By excluding these patients it allowed the evaluation of the CADILLAC risk score in predicting adverse events in stable patients presenting with a STEMI, and removing a subset of individuals who have significantly higher clinical event rates.

Utilization of scoring systems such as the CADILLAC risk score may carry clinical impact in patients presenting with a STEMI. Given upfront reperfusion with PPCI, mortality rates and MACE have been significantly reduced in patients presenting with a STEMI. The CADILLAC risk score appears to appropriately identify individuals who have a low risk of adverse cardiovascular events following reperfusion therapy with PPCI. Although not evaluated in this study, utilization of scoring systems such as CADILLAC may help to determine if all patients require the same level and duration of hospital care following revascularization. Currently practice guidelines recommend that most patients presenting with a STEMI spend at least 72 h in the hospital with the first 24 h under critical care monitoring. However, these current recommendations are predominantly based on data from the fibrinolytic era [9–12, 16, 17]. Given the practice shift away from fibrinolysis and towards PPCI, it may be possible for uncomplicated low risk STEMI patients to be safely discharged prior to the current recommended hospital course, or not require ICU monitoring. Several study have previously evaluated this, and demonstrated safe discharge prior to 72 h in uncomplicated STEMI patients [18–24]. Further studies dedicated to answering this clinically question would be needed to further assess safety of early discharge with utilization of the CADILLAC risk score.

## Limitations

This was a retrospective study performed in a single academic medical center. Attempted to replicate a previous study [14] to further validate and improve generalizability of the CADILLAC scoring system. Although our sample size was significantly larger than the previous study, the small sample size and low complication rate may be underpowered to detect small differences in outcomes between the low, intermediate, and high risk groups. Patients presenting with cardiogenic shock, cardiac arrest, or need for inotropic or mechanical support were excluded from the present trial. These factors would likely add additional incremental prognostic information and provide a true event rate for both the low and intermediate-high risk cohorts. However, given the high mortality rate and variability of care needed in these clinical situations, it was felt that including these patients would limit the studies reproducibility.

## Conclusion

Scoring systems, such as the CADILLAC risk score may help to risk stratify and identify patients whom are at low risk for complications following a STEMI. Patients presenting with a STEMI defined as low risk by the CADILLAC risk score were found to have lower event rates and no mortality events when compared to those with a CADILLAC score of 3 or greater. Additionally, having a low CADILLAC score was associated with a lower clinical event rate within the first year following their index hospitalization. This study further validates the CADILLAC score and its prognostic utility in identifying patients at low risk for adverse clinical events following STEMI.

## Data Availability

The datasets used and/or analyzed during the current study are available from the corresponding author (Ryan S. Wilson, MD) on reasonable request.
